# Cardiovascular abnormalities in multisystem inflammatory syndrome in children related to COVID-19

**DOI:** 10.3389/fped.2025.1635723

**Published:** 2026-01-05

**Authors:** Nathalie Jeanne Magioli Bravo-Valenzuela, Thiago Taucei Panizzi, Katharine Aguiar de Souza, Gabriela Blanco Stutz, Rafaela Vieira Meirelles Aurelio, Marta Cristine Felix Rodrigues, Rozana Gasparello de Almeida, Fernanda Maria Correia Ferreira Lemos, Alan de Lima Araújo, Flavio Roberto Sztajnbok, Adriana Rodrigues Fonseca

**Affiliations:** 1 Pediatrics Department, Instituto de Puericultura e Pediatria Martagão Gesteira/Federal University of Rio de Janeiro, Rio de Janeiro, Brazil; 2 Pediatric Cardiology Division, Instituto de Puericultura e Pediatria Martagão Gesteira/Federal University of Rio de Janeiro, Rio de Janeiro, Brazil; 3 Cardiology Institute of de Rio Grande do Sul, University Foundation, Rio Grande do Sul, Brazil; 4 Faculty of Medicine, Federal University do Rio de Janeiro, Rio de Janeiro, Brazil; 5 Pediatric Rheumatology Division, Instituto de Puericultura e Pediatria Martagão Gesteira/Federal University of Rio de Janeiro, Rio de Janeiro, Brazil

**Keywords:** COVID-19, Kawasaki, SARS-CoV-2, multisystem inflammatory syndrome in children, cardiovascular abnormalities

## Abstract

**Introduction:**

The COVID-19 pandemic began with the identification of SARS-CoV-2 in December 2019. Although children usually have milder acute symptoms, they can develop severe systemic symptoms termed pediatric multisystem inflammatory syndrome (MIS-C). This study reviews research in children and adolescents diagnosed with MIS-C, focusing on cardiovascular abnormalities.

**Methodology:**

This systematic review was conducted following PRISMA guidelines. The review protocol was prospectively registered in the Prospective Register of Systematic Reviews (PROSPERO; registration number: CDR420251232497). A search strategy was constructed to identify the studies focusing on cardiovascular abnormalities in children and adolescents with MIS-C published in Portuguese and English at PubMed and Scielo from January 2020 to February 2025. The eligibility criteria and data extraction strategy were guided by the PICO framework.

**Conclusions:**

Myocardial dysfunction and coronary abnormalities are the most frequent cardiovascular features in patients with MIS-C. Strain technology in echocardiography identifies early myocardial dysfunction, with studies showing persistent subclinical injuries. Despite ejection fraction and coronary anomalies returning to normal short to medium term, long-term cardiovascular effects of MIS-C remain uncertain, necessitating ongoing cardiology monitoring.

## Introduction

In December 2019, a new coronavirus variant responsible for a cluster of respiratory syndrome cases was described in China (1). Quickly coronavirus disease 2019 (COVID-19) was declared a pandemic by the World Health Organization (WHO) in March 2020 (2).

Clinical manifestations are varied and affect all age groups and can manifest mainly with fever, cough, dyspnea, and myalgia, gastrointestinal and skin changes (3).

Although initial pandemic research showed a lower propensity of children to develop more severe cases during the acute phase of the disease (4), severe cases have been increasingly reported in the literature as those with neurological manifestations (encephalopathy, stroke, Guillain-Barre) (5) and cardiovascular (heart failure, arrhythmias, myocarditis, pericarditis) (4, 6–9).

Patients with COVID-19 have a broad spectrum of cardiac clinical presentations. In the pediatric age group, some do not manifest clinical evidence of heart disease, but have abnormalities on cardiac tests (such as elevated serum cardiac troponin, asymptomatic cardiac arrhythmias, or abnormalities on cardiovascular imaging tests), and another group has symptomatic heart disease. Cardiac changes can occur in the acute phase or a few weeks after and included, (pericardial effusion), coronary artery vasculitis, valvulitis, heart failure (HF) and arrhythmias (4, 6–10).

In April 2020, reports from the UK documented a presentation in children of a Kawasaki-like disease or toxic shock syndrome or cytokine storm syndrome; these cases followed in 2–4 weeks the local acute COVID-19 spikes (2, 11, 12). Since then, there have been reports of similarly affected children in other areas of the world. The condition was named multisystem inflammatory syndrome in children (MIS-C) and in Brazil it is known as pediatric multisystem inflammatory syndrome (PMIS).

Kawasaki Disease (KD) is a vasculitis that affects preferentially the medium-sized arteries, especially the coronary arteries and, despite its similarity with MIS-C, aspects such as age of involvement and clinical spectrum may present differently (13, 14). Objectively, the World Health Organization (WHO) considers as defining a case of MIS-C (15):

- Individual aged 0–19 years presenting with fever for 3 or more days
- And two of the following criteria: 1. bilateral non-purulent cutaneous rash or conjunctivitis or signs of mucocutaneous inflammation (oral, hands or feet), 2. hypotension or shock, 3. Myocardial dysfunction, pericarditis, valvulitis, or coronary abnormalities (including ECO findings or elevated troponin/pro-BNP), 4. Evidence of coagulopathy (by elevated TAP, TTP, d-dimer), 5. Acute gastrointestinal problems (diarrhea, vomiting, or pain).
- And elevated markers of inflammation, such as erythrocyte sedimentation rate (ESR), C-reactive protein (CRP), or procalcitonin.
- And no other obvious microbial cause of inflammation, including bacterial sepsis, staphylococcal or streptococcal shock syndromes.
- AND evidence of COVID-19 (RT-PCR, antigen test, or positive serology) or history of contact with patients with COVID-19.

It has been suggested that MIS-C results from an abnormal immune response to the virus with some clinical similarities to Kawasaki disease (KD), macrophage activation syndrome (MAS) and cytokine storm syndrome. However, based on the available studies, MIS-C appears to have an immunophenotype distinct from KD and MAS (16, 17). Preliminary studies suggest that patients with severe MIS-C have persistent IgG antibodies with increased monocyte activation capacity, persistent cytopenias (mainly T-cell lymphopenia) and increased TCD8+ activation (16–19).

Considering that in the long term, the impact of MIS-C related myocardial injury is unknown, the present study aimed to review cardiovascular abnormalities in children and adolescents with MIS-C.

## Methods

### Electronic searches

This systematic review was conducted following PRISMA guidelines (Supplementary Table S1) and performed on 5 steps according to Khan KS et al. (20): 1- the review question, 2- study selection, 3- quality assessment of the studies, 4- data synthesis and 5- interpreting the findings (20). The search was based on the following question: “What are the cardiovascular changes related to Multisystem Inflammatory Syndrome in Children associated to COVID-19?”. The review protocol was prospectively registered in the Prospective Register of Systematic Reviews (PROSPERO; registration number: CDR420251232497).

A search strategy was constructed to identify the studies focusing on cardiovascular abnormalities in children and adolescents with MIS-C published in Portuguese and English at PubMed, Science Direct and Cochrane from January 2020 to February 2025. The Medical Subject Headings terms used were as follows: ‘COVID-19’ and ‘Multisystemic Inflammatory Syndrome in Children’ and ‘Kawasaki’ and ‘Cardiac involvement’. The eligibility criteria and data extraction strategy were guided by the PICO framework: (1) Population (P)- Children and adolescents (<21 years) diagnosed with Multisystem Inflammatory Syndrome in Children (MIS-C) temporally associated with SARS-CoV-2 infection according to CDC or WHO criteria, (2) Intervention/Exposure (I)-MIS-C clinical presentation and diagnostic evaluation, with emphasis on cardiovascular involvement assessed through biomarkers, electrocardiography, echocardiography, or cardiac MRI, (3) Comparison (C)-Not mandatory; when available, comparators included healthy pediatric populations, children with acute COVID-19, or children with Kawasaki disease and (4) Outcomes (O)- Cardiovascular manifestations (such as: ventricular dysfunction, coronary artery abnormalities, arrhythmias, pericardial involvement), treatment strategies, and short-term clinical outcomes. This PICO structure informed the selection of eligible studies, data extraction, and qualitative synthesis.

### Study selection

The titles and abstracts of all the publications obtained from the electronic search and the list of references of these studies were screened extensively by the authors. The pre-established inclusion and exclusion criteria were applied, and articles with complete available texts were selected to be examined and included in this review. Initially, the selected manuscripts were reviewed by six of the authors, and subsequently, they were verified by an additional five authors. Indeed, the studies selected were assigned to the quality of the research design such as homogeneous or heterogeneous populations, adjustment of bias factors, short or long-term follow-up and prospective or retrospective study. Any discrepancies were solved by consensus among the authors.

### Selection criteria

**Figure 1:**
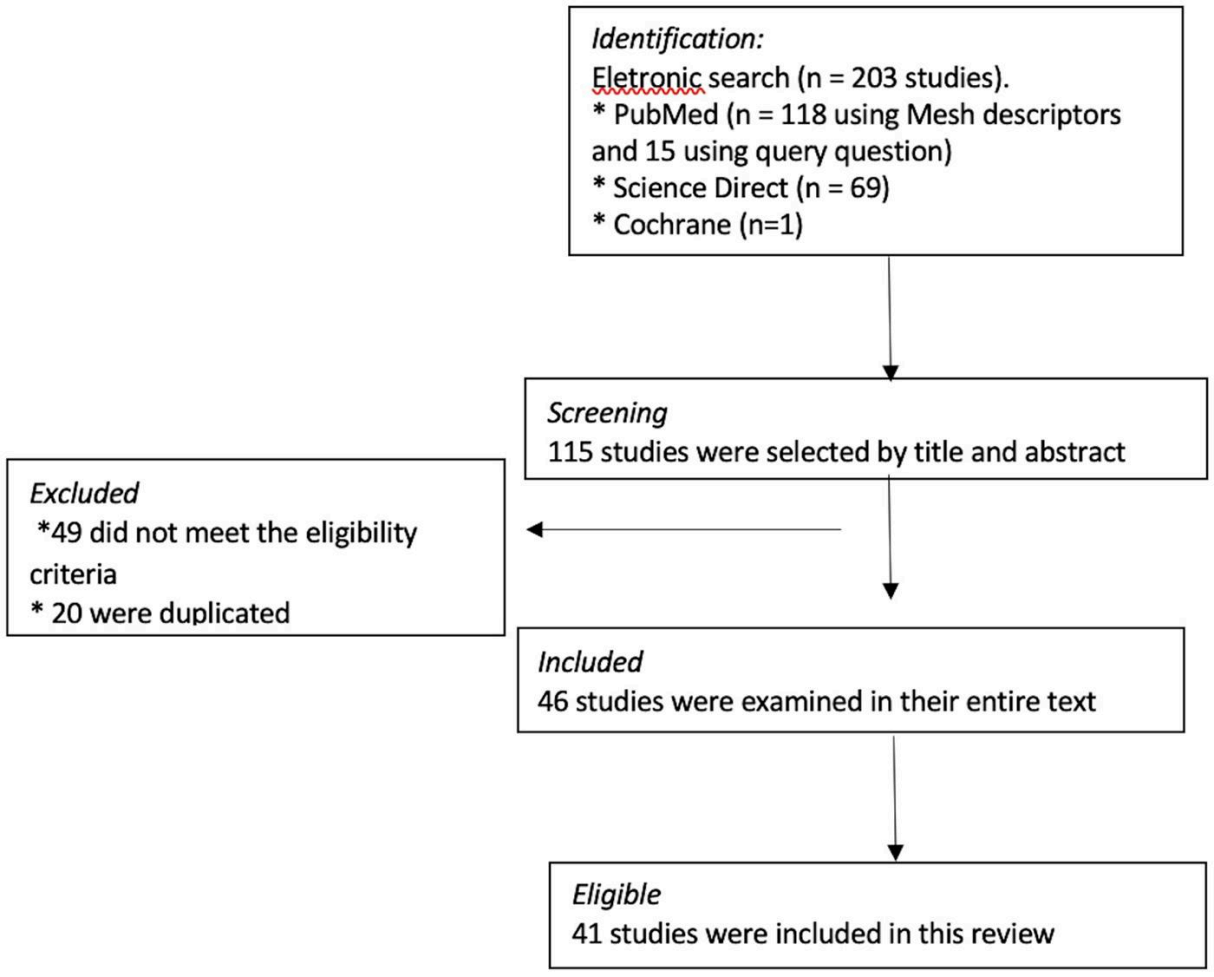
Flowcharts of records identified after an electronic search (by query question and descriptors) on multisystemic inflammatory syndrome in children and cardiac involvement performed from January 2020 to February 2025.

Case series reports, book chapters and review or prospective studies focusing on cardiovascular abnormalities in children and adolescents with MIS-C were selected. Criteria for inclusion of studies: 1-population: children and adolescents with MIS-C, 2- languages: Portuguese and English and 3-timeframe: from January 2020 to February 2025. A total of 115 related manuscripts were found after screening their titles and abstracts. After applying the inclusion and exclusion criteria, 46 manuscripts with complete available text were examined studies. After analyzing the selected studies and their references; overall, 41 studies were selected (Figure 1).

### Exclusion criteria

We excluded case reports (except for case series); duplicates; studies lacking full text; and studies not describing cardiovascular abnormalities in MIS-C.

## Results

### Selected studies

The electronic search retrieved a total of 203 records that were displayed based on the query question and mesh terms. Of these, 115 were selected, based on their title and abstract. Of them, 49 publications were excluded because they did not meet the selection criteria and 20 were duplicated. After analyzing them and their references, 46 were examined in their entire text, and 41 articles were considered eligible for the current review (Figure 1).

### Characteristics of the studies

Based on the studies included in this review, the main study designs are presented as follows: 12 retrospective studies, 1 ambidirectional study, 8 cross-sectional studies, 8 prospective observational cohort study (being 1 of them a case, a case-control study), 1 real-time survey, 4 systematic review/meta-analyses, 3 narrative reviews, 1 scoping review, 1 integrative review and 2 case series.

### Cardiovascular involvement in MIS-C

Current evidence suggests that approximately 75% of MIS-C patients develop cardiac dysfunction, 6%–24% present with coronary dilation, and 7%–60% demonstrate arrhythmias (4). Most affected children are previously healthy and school-aged, often presenting with features resembling Kawasaki Disease (KD). MIS-C accounts for a substantial proportion of pediatric COVID-19 mortality (6).

Compared with KD, MIS-C more frequently presents with shock. Early CDC data indicated that nearly half of affected children required intensive care with vasopressor support. Transient left ventricular dilation, systolic dysfunction, pericardial effusion, and mitral regurgitation are described, along with biomarkers indicative of myocardial injury (troponin, BNP/pro-BNP) and depressed ejection fraction. These findings may overlap with KD shock syndrome (21).

### Laboratory markers

Laboratory abnormalities correlate with disease severity (21–23). Reported changes include lymphopenia, neutrophilia, anemia, thrombocytopenia, elevated CRP, ESR, liver enzymes, and cardiac biomarkers (1, 2, 4, 6–9, 13, 14, 16, 17, 24).

### Mechanisms of injury

Cardiac injury in MIS-C appears multifactorial, involving systemic inflammation, acute viral myocarditis, hypoxia, stress cardiomyopathy, and ischemia due to coronary involvement (4). Several mechanisms may contribute simultaneously (23).

### Age-related features

Bulut et al. reported that myocardial dysfunction and coronary abnormalities are the most common cardiovascular manifestations in MIS-C, with higher prevalence among males >10 years (25). Campanello et al. demonstrated age-dependent patterns: children <6 years more frequently exhibited coronary dilation or aneurysms, while older children showed more myocardial dysfunction and pericardial involvement (26).

### Disease severity

In a meta-analysis of 318 pediatric cases, Toraih et al. identified KD-like features in most patients, with 68.1% progressing to shock and 41.1% developing acute kidney injury, reinforcing the need for cardiac and renal monitoring (27). Abrams et al. identified risk factors for severe disease, including age >5 years, elevated ferritin, and elevations in troponin, BNP, or pro-BNP (23).

### MIS-C and Kawasaki disease (KD)

MIS-C and KD have overlapping manifestations but present key differences. KD typically affects younger children, whereas MIS-C presents with hyperinflammation, hypercoagulability, circulatory shock, coronary vasculitis, myocardial dysfunction, and pericarditis (28). Although both may cause coronary dilation or aneurysm, these findings appear more common in KD (28). Whittaker et al. reported more severe MIS-C, with pronounced myocardial injury and coronary aneurysm (22). Narrative reviews showed that cardiac involvement is substantial in MIS-C, including coronary injury and reduced contractility (29, 30). Kabeerdoss et al. demonstrated that KD and KD-like MIS-C are distinct clinical entities; symptomatic myocarditis occurred in 40%–80% of MIS-C cases but <5% of KD cases. NT-pro-BNP and troponin were markedly elevated in MIS-C. A meta-analysis showed higher coronary abnormality rates in MIS-C compared with KD (40.8% vs. 14.8%) (31).

### Recovery and follow-up

Most patients show recovery of ventricular function and normalization of biomarkers within three to six months. Studies by Mannarino, Uygun, and others highlight excellent short-term recovery; however, patients with coronary aneurysms require continued follow-up (32, 33). McAree et al. demonstrated reduced exercise capacity six months post-hospitalization, suggesting potential subclinical impairment and the need for long-term surveillance (34).

Multiple studies (35–37) confirmed rapid improvement of cardiovascular abnormalities despite initial severity. Webster et al. and Zimmerman et al. reported normalization of cardiac biomarkers and MRI within two months (38, 39). Minocha et al. found normalization of cardiac testing after discharge in 73% of children with abnormal admission studies (40). Subsequent reports confirmed recovery of function at 6–12 months (36, 41, 42). A 2023 meta-analysis demonstrated that most children regained normal ventricular function within three months, although coronary abnormalities or mitral regurgitation occasionally persisted up to six months (43).

### Cardiac imaging

Echocardiography is the cornerstone imaging modality for MIS-C. Left ventricular dysfunction affects ∼38% of patients and is associated with increased mortality (9.5% vs. 1.5%) (44). Troponin, BNP, and CRP correlate with severity (44, 45). Approximately 70% of affected children show ECG abnormalities, including low QRS voltage and transient anterior T-wave inversion (46).

Speckle-tracking echocardiography improves detection of subtle dysfunction; strain values remain reduced compared with controls during follow-up (47).

Cardiac MRI is valuable for assessing myocardial inflammation, edema, or perfusion abnormalities (48, 49). Early abnormalities are common, but recovery is typically rapid. Karagözlü et al. and Aeschlimann et al. reported normal follow-up MRI in most children despite initial dysfunction (49, 50). Chakraborty et al. demonstrated recovery of left ventricular function and coronary edema at 3–6 months (51).

In a multicenter cohort, De Wolf et al. reported excellent late outcomes when patients were managed per guidelines; MRI demonstrated no scarring in children with normal systolic function. However, reduced global longitudinal strain persisted in a subset, suggesting subclinical dysfunction (52). Phirtskhalava et al. e Shah et al. and similarly reported complete recovery with corticosteroids and/or IVIG, though long-term data remain limited (53, 54).

More recently, Anagnostopoulou et al. observed abnormal global longitudinal strain despite preserved ejection fraction during follow-up, underscoring the need for continued surveillance (55). Similar findings from Sabri et al. and Leal et al. reinforce the diagnostic value of strain imaging (47, 56).

Supplementary Table S2 illustrates the studies included in the current review.

## Limitations

MIS-C is a rare disease of recent onset. Consequently, current studies in the existing literature lack long-term follow-up. Another factor that must be considered is the heterogeneity of the study design and sample size. In addition, this is a systematic review, and no statistical analysis was carried out on the data collected.

## Conclusions

MIS-C is associated with significant cardiovascular involvement, most commonly myocardial dysfunction and, less frequently, coronary abnormalities (KD-like presentation). MRI is useful in evaluating myocardial inflammation and guiding follow-up, while echocardiography is crucial for evaluating coronary arteries and myocardial function during acute MIS-C. Although ventricular function and cardiac biomarkers typically normalize within months, subclinical myocardial dysfunction may persist, particularly detectable by strain imaging. Strain technology in echocardiography offers early detection of myocardial dysfunction, with studies showing lower strain values and subclinical myocardial injuries persisting in some patients. Because the long-term cardiovascular consequences of MIS-C remain uncertain, ongoing cardiologic surveillance is recommended, particularly for patients with coronary abnormalities or impaired strain values at follow-up. Future prospective studies with longer follow-up are needed to clarify the temporal evolution of subclinical myocardial abnormalities and potential long-term risks.

## Supplementary material

Supplementary Material Table S1 PRISMA 2020 Checklist – Systematic Review.

Supplementary Material Table S2 Studies included in this review.
