# Supplementary material for: Cardiovascular abnormalities in multisystem inflammatory syndrome in children related to COVID-19
Source: Front Pediatr. 2026 Jan 5;13:1635723. doi: 10.3389/fped.2025.1635723 (PMC12812880; doi:10.3389/fped.2025.1635723)
Supplement: Supplementary Material Table S1 — PRISMA 2020 Checklist – Systematic Review. [file Table1.docx]

| **_AUTHOR/YEAR/JOURNAL/REF._** | **_SAMPLE (N)_** | **_STUDY DESIGN_** | **_CHARACTERISCTICS OF THE POPULATION_** | **_CONCLUSIONS_** |
| --- | --- | --- | --- | --- |
| _Riphagen S et al, 2020_  _The Lancet (2)_ | _8_ | _Case series_ | _- 8 children with hyperinflammatory shock, age: 4 to 14 years (mean: 8.8 years), 5 boys and 3 girls._  _- Ethnicities included: 6 children of Afro-Caribbean descent, 1 Middle Eastern, and 1 Asian._  _-All children were previously > 75th centile for weight, except 1._  _- 4 children had known family exposure to coronavirus disease 2019 (COVID-19)._ | _- The research indicates that children who were previously without symptoms but have contracted SARS-CoV-2, showing characteristics of a hyperinflammatory syndrome similar to Kawasaki disease shock syndrome._  _- Given the intricate nature of this condition, collaborative care from diverse specialties becomes crucial._  _- This study aims to highlight the importance aim of early identification and optimal treatment for this group of children in the pediatric community._ |
| _Rodriguez-Gonzalez M et al, 2020_  _World Journal of Clinical Cases (6)_ | _16 studies with over 10 patients_ | ^Systematic review and meta-analysis^ | _-Of 193 articles, this review focused on 16 studies with over 10 patients, providing comprehensive data on cardiovascular involvement in pediatric multisystem inflammatory syndrome (PMIS)._  _- Additionally, 10 articles reported isolated cases of cardiovascular issues in previously healthy children, and 10 studies documented cases in children with pre-existing heart conditions_  _-The meta-analysis of the 16 studies highlighted a predominant impact on previously healthy school-aged children and adolescents with MIS-C, often presenting with KD-like features and multiple organ failure, emphasizing that these cases accounted for most of pediatric mortality from COVID-19._  _- 10 articles reported sporadic instances of cardiovascular complications in previously healthy children, while another 10 studies documented cases in children with pre-existing heart diseases. Most cases involved severe COVID-19 infections with full recovery following intensive care support, but instances of mortality were also identified._ | _- Limited data exists regarding the role of cardiovascular involvement in COVID-19 among children._  _- Based on the review, children, whether previously healthy or with pre-existing heart conditions, should undergo a cardiac evaluation and receive close cardiovascular monitoring if hospitalized for acute COVID-19._ |
| _Valverde I et al, 2021_  _Circulation (7)_ | _286_ | _Real-time internet-based survey_ | _-Children with MIS-C, median age: 8.4 years, 194 (67.8%) boys, ethnicity: 161 white, 59 black, 29 Asian, 17 mixed, and 20 classified as other._  _-Most of children were previously healthy, with only 4 having CHD and 12 having an associated autoimmune disorder._  _-Most families were reported as healthy, but 14.7% of family members tested positive for SARS-CoV-2._ | _-Cardiac participation is frequently observed in pediatric cases of multisystem inflammatory syndrome linked to the Covid-19 pandemic._  _- Most children exhibit notably elevated levels of N-terminal pro B-type natriuretic peptide, ferritin, D-dimers, and cardiac troponin, alongside heightened levels of C-reactive protein and procalcitonin._  _- Despite the multisystem impact, extremely elevated inflammatory markers, and the necessity for intensive care support, mortality in children with multisystem inflammatory syndrome associated with COVID-19 is infrequent when compared to adults with COVID-19._ |
| _Belhadjer Z et al, 2020_  _Circulation (8)_ | _35_ | _Retrospective multicenter study_ | _- 35 children who were admitted in pediatric IUC (14 centers in France and Switzerland) with cardiogenic shock, LV disfunction and severe inflammatory state_  _- Median age at admission: 10 years (2-16 years), 18 males and 17 females._  _-Comorbidities: 28% of the cases, none of them had underlying cardiac disease and 17% with overweight._  _- 31 (88.5%) tested positive for SARS-CoV-2 and 13 with history of recent contact with family members displaying virus-like symptoms._ | _- Following SARS-CoV-2 infection, children may undergo acute cardiac decompensation due to a severe inflammatory state, known as multisystem inflammatory syndrome in children._  _-_  _Pediatric and cardiology communities should be aware of this new disease probably related to SARS-CoV-2 infection (MIS-C) that shares similarities with Kawasaki disease but has specificities in its presentation._  _- Administration of immunoglobulin seems to be linked with the restoration of left ventricular systolic function, suggesting a potential therapeutic benefit in such cases._ |
| _Alonso I de AF et al, 2021_  _Brazilian Journal of Development (9)_ | _427_ | ^Integrative review^ | _- 20 selected articles included data from 427 pediatric patients who contracted SARS-CoV-2 and subsequently developed MIS-C, possibly linked to the emergence of cardiac diseases._ | _-Findings revealed a spectrum of cardiovascular issues post-COVID-19, including reduced left ventricular ejection fraction, vasculitis, mild mitral insufficiency, coronary artery dilation, pericardial effusion, valvular regurgitation, myocarditis, and electrocardiographic changes._  _-The results suggest severe forms of the disease can develop after the immune response o SARS-CoV-2, challenging the notion that COVID-19 has no adverse effects on children. - This underscores the need for further exploration by clinicians and researchers to better understand the disease._ |
| _Mileva N et al, 2023_  _Medicina (10)_ | _51 children_ | _Cross-sectional_ | _- 51 patients meeting the MIS-C criteria_ _according to the CDC criteria, from 25 November 2020 to 24 April 2021._  _- 14 (27.5%) of the MIS-C patients had the following comorbidities: drug allergies, allergic rhinitis, mild bronchial asthma, cerebral palsy, and epilepsy._ | _- Children with oligosymptomatic MIS-C or those suspected of long COVID-19 with elevated IL-6 and other inflammatory activity, should be screened for possible cardiological involvement_ |
| _Carter MJ et al, 2020_  _Nature Medicine (16)_ | _25_ | _Prospective cohort_ | _- 25 patients of Evelina London Children’s Hospital, recruited during the pandemic, median age: 12.5 years, 10 (40%) females, and 10 (40%) were identified as of white ethnicity._  _- 17 patients (68%) tested seropositive for SARS-CoV-2-specific antibodies, 1 patient tested positive for SARS-CoV-2 by PCR with reverse transcription (RT-PCR)._  _- Among 8 seronegative children (32.0%): 6 had history of previous symptoms indicative of SARS-CoV-2 infection, close household contact with confirmed cases, attendance at mass gatherings, or parents who were healthcare workers._ | _- MIS-C is likely an independent immunopathogenic condition linked to SARS-CoV-2, showing a tendency for more severe illness in seropositive children._  _- Investigating the mechanisms that underlie these immune abnormalities is crucial and warrants further research attention._ |
| _Lee PY et al, 2020_  _Clinical Investigation (17)_ | _28_ | _Cross-sectional_ | _- median age: 9 years, 57% being male, 18% were Black, and 43% were Hispanic._  _-14 patients (50% of the cohort) had preexisting medical conditions: obesity (n = 4), asthma (n = 3), congenital heart disease (n =1), sickle cell anemia (n =1), mitochondrial disorder (n = 1), autism (n = 1), and chromosomal abnormalities (n = 1)._  _- History of KD: 2 patients, 1 of whom had coronary aneurysm._  _-Confirmatory evidence of SARS–CoV-2 infection: all patients_ | _- MIS-C linked to COVID-19 represents a hyperinflammatory syndrome characterized by a wide range of clinical manifestations and varying degrees of severity._  _- While sharing certain features with inflammatory processes observed in Kawasaki disease and macrophage activation syndrome (MAS), MIS-C also exhibits distinct characteristics._  _- Swift diagnosis, a multidisciplinary approach to management, and the reduction of systemic inflammation are correlated with positive outcomes in the majority of patients._ |
| _Whittaker E et al, 2020_  _JAMA (22)_ | _58_ | _Case series (cohort study)_ | _-58 hospitalized children (8 hospitals in England from March to May, 2020) with criteria for MIS-C associated to SARS-Cov-2_  _-Median age: 9 years, females: 34% of the cohort._  _- 45 patients (78%) exhibited evidence of current or prior SARS-CoV-2 infection._ | _-This case series showed a variety of signs, symptoms, and disease severity in hospitalized children who criteria for MIS-C._  ^- Manifestations ranged from fever and inflammation to myocardial injury, shock, and the formation of coronary artery aneurysms^  -_Comparing patients with Kawasaki disease (KD) and KD shock syndrome reveals key differences from other pediatric inflammatory conditions._ |
| _Abrams JY et al, 2021_  _Lancet Child Adolescent Health (23)_ | _1080_ | _Retrospective cohort_ | _-602 patients (56%) were male, with a median age of 8 years and 286 (26%) had obesity._  _-The majority, 724 out of 945 patients (77%), identified as either Hispanic or non-Hispanic Black._ | _-The clinical presentations in children with MIS-C exhibit variability._  _- Age beyond 5 years and specific laboratory indicators, such as troponin, BNP, proBNP, ferritin, C-reactive protein, and D-dimer concentrations, can serve as valuable markers for identifying those at an elevated risk of experiencing severe disease outcomes._  _-These outcomes may include ICU admission, shock, and compromised cardiac function._  _-Recognizing the characteristics associated with an increased risk of severe outcomes could contribute to better-informed management strategies for children hospitalized with MIS-C._ |
| _Bulut M et al, 2023_  _Turk Archives of Pediatrics (25)_ | _38_ | _Single center,_  _retrospective observational study_ | _-Patients with MIS-C, mean age: 9.6 years_ _and 23 patients (60.5%) were of male sex._  _-Comorbid diseases were found in 7 cases (18.4%) but none had preexisting heart disease._  _-Cardiovascular involvement occurred in 25 cases (65.7%)_ | _- Cardiovascular abnormalities in patients with multisystem inflammatory syndrome show rapid resolution within the first month._  _- Myocardial dysfunction and coronary abnormalities are the most prominent features in patients with MIS-C._  _- Clinicians should suspect cardiovascular involvement in male patients older than 10 years, especially in severe clinical courses._  _- Echocardiographic abnormalities exhibit swift resolution within 6 months._  _-However, it is crucial to note that coronary aneurysms require regular long-term follow-up._ |
| _Campanello C et al, 2022_  _Children (26)_ | _25_ | _Cross-sectional_ | _-25 children diagnosed with MIS-C, with a median age of 5 years._  _- The cohort was divided into 2 groups: 13 children under 6 years old (52%) and 12 aged 6 years or older (48%), with an equal distribution between males (56%) and females (44%)._ | _- MIS-C presents age-dependent cardiovascular manifestations._  _- Children under 6 years old predominantly exhibited coronary abnormalities, such as dilatations and aneurysms, while older children showed a higher prevalence of myocardial dysfunction and pericardial involvement._  _- Early and aggressive anti-inflammatory treatment led to favorable outcomes, with recovery of cardiac function and resolution of most coronary anomalies by discharge._ |
| _Toraih EA et al, 2021_  _World Journal of Pediatrics (27)_ | _318_ | _Meta-analysis_ | _-Studies on pediatric COVID-19 cases, median age: 9,1 years, combined prevalence of boys across the studies: 50.5%._  _- The proportion of Black patients was 36.8%, which was higher compared to the white cohort (16.4%) and the Asian cohort (13.6%)._ | _- Identifying both the usual and unusual manifestations of multisystem inflammatory syndrome in pediatric COVID-19 cases holds significant importance for recognizing children at risk._  _-Vigilant monitoring of cardiac and renal decompensation, coupled with prompt interventions, is crucial in patients with multisystem inflammatory syndrome to avoid additional morbidity._ |
| _Mavrogeni SI et al, 2021_  _Rheumatology International (29)_ | _Not informed_ | _Narrative review_ | _- This narrative review included manuscripts published from March to December 2020._  _-Databases: Medline, Embase, Scopus_ | _- ECG abnormalities were found in 56% of MIS-C patients._  _- Echocardiography is the cornerstone modality for myocardial function and coronary artery evaluation in the acute phase._  _- Cardiovascular magnetic resonance detects diffuse myocardial inflammation including oedema/fibrosis, myocardial perfusion and coronary arteries anatomy during the convalescence and in adolescents, where echocardiography may provide inadequate images._  _-Cardiac biomarkers including N-terminal pro b-type Natriuretic Peptide (NT-pro-BNP) and troponin levels are extremely high, compared to KD and indicate severe myocardial damage that may lead to heart failure._  _•-Symptomatic myocarditis was found in 40–80% of patients with MIS-C._  _-In contrast, symptomatic myocarditis is seen in < 5% of KD patients._  _- Coronary artery abnormalities have been diagnosed in 9–24% of patients with MIS-C, as common as in KD in the intravenous immunoglobulin era._ |
| _Kabeerdoss J et al, 2021_  _Rheumatology International (30)_ | _Not informed_ | _Narrative review._ | _- A search of the literature was conducted using Medline/Pubmed, Scopus, and Embase to explore COVID-19 in children, multisystem inflammatory syndrome in children, and Kawasaki disease for the timeframe from December 1, 2019, to August 31, 2020._ | _- MIS-C is a hyper-inflammatory syndrome affecting multiple organs, triggered by SARS-CoV-2 infection, typically appearing 2–4 weeks post-infection._  _• Adaptive immune mechanisms significantly contribute to its pathogenesis._  _• While MIS-C and Kawasaki Disease share some clinical manifestations, they are considered two distinct conditions._ |
| _Arantes Junior MAF et al, 2023_  _Reviews in Medical Virology (31)_ | _1522_ | _Systematic review and meta‐analysis_ | _-Systematic review and meta‐analysis, examined the pooled prevalence of cardiovascular manifestation and cardiac complications in children hospitalized with MISC._  _- Articles published between March 2020 and May 2022 were included._ | _- The combined prevalence of myocarditis or pericarditis was 34.3%. The combined prevalence for echocardiogram anomalies was 40.8%, that of Kawasaki disease presentation was 14.8%, and that of coronary dilation was 15.2%. The rate of electrocardiogram anomalies was 5.3%, and the mortality rate was 0.5%._  _-Studies that assess whether these children will have an increased cardiovascular risk with a greater chance of acute myocardial infarction, arrhythmias, or thrombosis will be essential for healthcare planning_ |
| _Mannarino S et al, 2022_  _Italian Journal of Pediatrics (32)_ | _32_ | _Cross-sectional_ | _- 32 children with MIS-C, median age: 10 years, 75% were male, 81% had cardiac involvement, and 69% required pediatric ICU admission._ | _-Significant cardiac involvement was observed but all patients recovered completely with appropriate treatment._  _-Factors such as elevated CRP, troponin, and NT-pro BNP were associated with more severe cardiac dysfunction._ |
| _Uygun H et al, 2024_  _The International Journal of Cardiovascular Imaging (33)_ | _93 patients (38 MISC and 55 controls)_ | _Case-control_ | _- 38 patients in the experimental group, median age: 9.05 years; 24/38 (63%) were male and 14/38 (37%) were female._  _- 55 patients in the control group, median age: 9.00 years; 36/55 (65%) were male, and 19/55 (35%) were female._  _- Median height was 132 cm in the experimental group and 137 cm in the control group. Mean BMI was 18.96 in the experimental group and 18.95 in the control group._ | _-Many pathological cardiovascular findings detected at diagnosis, such as mitral valve insufficiency and left ventricular dysfunction, improved significantly within six months._  _-Aortic stiffness parameters were also evaluated, showing partial normalization over time._ |
| _McAree D et al, 2023_  _Pediatric Cardiology (34)_ | _22_ | _Retrospective single-center cohort_ | _- 22 MIS-C patients, with a mean age of 11.9 years. Among the group, 81.8% were male._  _-Exercise stress testing (EST) was performed approximately 6 months post-hospitalization, and results were compared to 33 myocarditis patients (mean age 15.5 years) and 44 healthy controls (mean age 12.0 years)._ | _- The study concluded that MIS-C patients experience significantly reduced exercise capacity and aerobic performance at six months post-hospitalization compared to myocarditis patients and healthy controls._ |
| _Wu EY et al, 2021_  _Current Cardiology Reports (35)_ | _Not informed_ | _Narrative review_ | _- Manuscripts on cardiac manifestations and treatments of MIS-C associated with COVID-19_  _-Cardiac involvement occurs in up to 67–80% of children with MIS-C. Prevalence of coronary artery aneurysms in MIS-C: approximately 13–26% (often mild), more common in male patients and in those with mucocutaneous and conjunctival involvement._  _-Ventricular dysfunction is a common finding with 33–50% of patients affected. A reduction in LVEF is the most reported finding (34–50% of children). Areas of hyperenhancement are uncommon on late gadolinium enhancement, occurring in up to 14% of patients._  _- ECG findings: arrhythmias and conduction abnormalities (28–67% of MIS-C patients), low QRS amplitude and T-wave abnormalities._ | _- Cardiac manifestations are common and occur on a spectrum of severity._  _- Current treatment strategies have proven effective at resolving many of these cardiac findings, but there is still room for improvement._  _- Close disease surveillance is ongoing and will further characterize the cardiac manifestations and potential sequelae of MIS-C._  _- Multi-center collaborations and harmonized registries are key to understanding the natural history, refining diagnostic criteria, developing risk stratification algorithms, and determining best management._ |
| _Kapoor R et al, 2023_  _Indian Journal of Pediatrics (36)_ | _54_ | _Retrospective observational study_ | _-54 children admitted to a hospital in India from September 30, 2020, to June 6, 2021._  _-Median age: 5.5 years (IQR 8.75) Of these, 37 (68.5%) were male._ | _- MIS-C poses a serious complication after COVID-19 in children, with cardiac involvement that typically resolves over time._  _•-Follow-up revealed no long-term cardiac sequelae, highlighting that, although severe at onset, MIS-C can be managed effectively with intensive care and immunomodulation_ |
| _Jaxybayeva I et al, 2023_  _Acta Biomedica (37)_ | _93_ | _Retrospective observational study_ | _-Children who had MIS-C with mean age of 6 years, comprising 65 boys (69.9%) and 28 girls (31.1%)._  _- Follow-up duration varied, with 43 children (46.4%) observed for over 2 years, 42 (45.1%) for 1 to 2 years, 6 (6.4%) for 6 months to 1 year, and 2 (2.1%) for up to 6 months._ | _- Despite the severity of the disease, most children experienced minimal long-term consequences after recovery._  _-The recurrence rate was 2.2%, with complications such as somatic disorders (11.8%), anemia (8.6%), decreased vision (7.5%), and reactive arthritis (2.2%)._  _- Coronary artery dilation significantly decreased, with only one case (1.7%) remaining. Although echocardiograms showed a reduction in cardiovascular issues, some children exhibited increased tricuspid and pulmonary regurgitation, without full restoration of cardiovascular function._ |
| _Webster G et al, 2021_  _J Cardiovascular Magnetic Resonance (38)_ | _11 COVID19, 6 MISC,_  _29 controles_ | _Prospective cohort_ | _-Prospectively recruited pediatric cohort recovered from COVID-19 and MISC, age range: from10 to 18years, from Sept to Dec 2020, 2 months after recovery._  _- Exclusion criteria: children with pre-existing cardiac disease were excluded._ | _-Children prospectively recruited following SARS-CoV-2 infection had normal CMR and cardiac biomarker evaluations during mid-term recovery._ |
| _Zimmerman D et al, 2023_  _Pediatrics (39)_ | _69_ | _Single-center retrospective study_ | _-69 children diagnosed with MIS-C, with a mean age of 10 years and 64% male._  _-Among the patients, 54% had evidence of myocardial injury. Coronary artery abnormalities were present in 38% during hospitalization, with dilation and aneurysms being the most frequent findings._ | _- The study found a high prevalence of abnormal findings in cardiac follow-up studies, regardless of whether patients had myocardial injury during acute illness._  _-Abnormalities included elevated extracellular volume, late gadolinium enhancement, and reduced functional capacity in cardiopulmonary exercise testing._  _-These findings suggest that subclinical cardiovascular pathology may persist even in those without apparent initial myocardial injury._ |
| _Minocha PK et al, 2021_  _Clinical Pediatrics (40)_ | _33_ | _Retrospective, observational study_ | _-Patients aged 21 years and younger who were hospitalized with MIS-C between March 1, 2020, and June 8, 2020._  _- Exclusion criteria: patients with acute respiratory COVID-19 infection_  _- Median age: 2.8 years, with male predominance (58%) and Hispanics constituting the largest ethnic group (36%)._  _- Comorbidities included: obesity (21%) and asthma (15%)._ | _- Of the 33 patients in the study cohort, 24 (73%) had at least one abnormality in cardiac testing: abnormal electrocardiogram (48%), elevated brain natriuretic peptide (43%), abnormal echocardiogram (30%), and/or elevated troponin (21%)._  _- Mild or moderate regurgitation was seen in 4 (13%) patients, while 2 (7%) patients had mild left main coronary artery (LMCA) dilation with z scores of +2.3 and +2.7._  _- Electrocardiogram and echocardiogram abnormalities all resolved by the 2-week outpatient follow-up cardiology visit._  _- A CRP >50 mg/L and BNP >100 pg/mL were shown to have a statistical association with abnormal echo findings in our study._  _- Cardiac dysfunction resolved completely in all patients and normalized within a short timeframe as reported at other centers._ |
| _Garbin M et al, 2022_  _Children (41)_ | _32_ | _Retrospective, single-center study_ | _-32_ _patients with MIS-C were enrolled and underwent advanced echocardiogram at discharge and at 6 months._  _- 2 groups: group A, patients with moderate to severe depression of LVEF (LVEF < 45%), and group B, patients without or with mild signs of cardiac involvement (LVEF 45%)._ | _- MIS-C is a rare and potentially fatal novel disease, in which the \heart and the cardiovascular system are frequently involved._  _- Despite significant cardiac dysfunction in the early stage, all the traditional and advanced echocardiographic parameters were normal at discharge and at 6 months follow-up._ |
| _Sanil Y et al, 2021_  _Journal of the American Society of Echocardiography (42)_ | _54_ | _Prospective Cohort_ | _- Cohort of 54 patients with MIS-C ( mean age of 6.8 years), of whom 46% (n = 25) were male and 56% (n = 30) were African American._ | _- Median left ventricular apical four-chamber peak longitudinal strain (LVA4LS) and left ventricular global longitudinal strain (LVGLS) at initial presentation were significantly decreased in patients with MIS-C compared with the normal cohort (16.2% and 15.1% vs 22.3% and 22.0%, respectively)._  _- LVA4LS and LVGLS were able to indicate the risk for subclinical LV dysfunction persistent up to 10-week follow-up after resolution of acute illness in a subset of patients with MIS-C, independently of other echocardiographic parameters and inflammatory biomarkers._ |
| _Yasuhara J et al, 2023_  _Pediatric Cardiology (43)_ | _547_ | _Systematic review and meta-analysis_ | _-Studies on the follow-up ranged from 3 months to 1 year_  _-Patients were from multiple observational studies_ | _-Most children with MIS-C experienced recovery of left ventricular systolic dysfunction by 3 months._  _- However, coronary abnormalities persisted in a small proportion (5.2%) of patients at 6 months._  _- Mitral regurgitation also persisted in 7.5% of patients at 6 months._ |
| _Phung NTN et al, 2022_  _Pediatrics and Neonatology (44)_ | _76_ | _Retrospective cohort study_ | _-Children admitted to Children's Hospital in Ho Chi Minh City from September 1, 202 to February 28, 2022._  _-Median age: 5.9 years, with a male-to-female ratio of 1.6:1. Only one child had a pre-existing medical condition._ | _-The study found significant cardiovascular involvement in children with MIS-C, including coronary injury and reduced myocardial contractility, though treatment with IVIG and methylprednisolone yielded positive short-term outcomes._  _-The absence of fatalities and a mean hospitalization duration of 7.2 days suggest effective acute management, but long-term follow-up is recommended to understand potential sequelae and optimize treatment approaches​_ |
| _Atasayan V et al, 2023_  _Cardiology in the Young (45)_ | _67_ | _Cross-sectional_ | _- 67 children hospitalized with a diagnosis of MIS-C, 74.6% were boys, with an average age of 8.5 years_ | _- Elevated inflammatory markers such as BNP, troponin, and the neutrophil-to-lymphocyte ratio were associated with systolic dysfunction. The mortality rate was 1.5%._ |
| _Broberg MCG et al, 2023_  _Annal Academy Medical_  _of Singapure (46)_ | _Not informed_ | _Scoping Review_ | _-Scoping review of cardiovascular disease associated with COVID-19_ | _-Approximately 70% of patients with MIS-C have ECG changes,_  _-Left ventricle dysfunction is common with a pooled prevalence of 38%, with both global dysfunction and regional wall motion abnormalities described._  _- Troponin, BNP and CRP levels correlate with ventricular dysfunction severity._  _- Mild or moderate atrioventricular regurgitation is seen in 10–72% of patients._  _-Global circumferential strain, peaklet atrial strain, and peak longitudinal strain of the RV free wall are predictive of myocardial injury._  _- Most patients with left ventricle dysfunction recover within 30 days; however, diastolic dysfunction may persist._ |
| _Sabri MR et al, 2025_  _Pediatric Cardiology (47)_ | _29 MIS-C and 29 controls_ | _Prospective, longitudinal study_ | _-Pediatric patients with MIS-C hospitalized at Hossein Children’s Hospital from late 2021 to early 2022 were included and prospectively followed._  _- Mean age: 5.1 years_  _- 29 of them, comprising 14 males (48.3%) and 15 females (51.7%), underwent STE and were compared with 29 healthy age- and sex-matched._ | _- Global strain rate (GLS) and strain rates were not significantly different but were still lower than the control group._  _- Twenty percent of patients had abnormal GLS but normal left ventricular ejection fraction (LVEF)._  _- All patients exhibited reduced segmental myocardial strain in at least one segment._  _- Four out of 26 recovered patients without comorbidities had abnormal GLS at follow-up, despite normal LVEF._  _- STE proves more useful than conventional echocardiography in patients with MIS-C, revealing subclinical cardiac injury in the acute and post-acute phases._ |
| _Krupickova S et al, 2022_  _The International_  _Journal of Cardiology (48)_ | _60 (30 MIS-C and 30 controls)_ | _Cross-sectional_ | _-Retrospective study 30 following MIS-C and 30 controls. Strain values were compared between patients and controls and additionally to published pediatric normal CMR values._  _-Median age of the patients was 9.0 years and controls 9.8 years._ | _-Short-term sequelae of Multisystem Inflammatory Syndrome in Children (MIS-C) showed rapid improvement of the cardiac abnormalities within a few weeks after the onset of the disease. - However, subtle residual abnormalities, affecting mainly the myocardial interstitium, were shown in some of the patients_  _-All conventional CMR parameters in patients were in normal range_  _- Strain values were significantly lower in patients than in controls_ |
| _Karagözlü S et al, 2024_  _Cardiology in the Young (49)_ | _44_ | _Prospective longitudinal (0bservational descriptive study)_ | _-44 children diagnosed with MIS-C with cardiovascular involvement._  _-The mean age was 8.5 years, with 56.8% being male. respiratory support and 40% needing inotropic support._ | _-Myocardial involvement is common during the acute phase of MIS-C but resolves in most cases within one year with appropriate treatment._  _- Cardiac magnetic resonance imaging (MRI) detected myocardial inflammation in some cases, but all findings normalized during follow-up._  _-Elevated NT-proBNP levels correlated with disease severity and left ventricular systolic dysfunction, proving valuable for assessing cardiac impact._  _- Cardiac MRI remains an important tool, especially for detecting subtle abnormalities like fibrosis and edema, even in cases with normal echocardiography, supporting its use in selected follow-up scenarios._ |
| _Aeschlimann FA et al, 2021_  _Journal of Cardiovascular Magnetic Resonance (50)_ | _111_ | _Retrospective, observational, international multicenter cohort_ | _-111 patients who met criteria for MIS-C having cardiac involvement and underwent cardiovascular CMR._  _-Median age at disease onset was 10.0 years and comorbidities were reported in 25/106 (24%) children, most commonly respiratory diseases such as bronchiolitis or asthma. None of the patients had associated pre-existing cardiac pathologies._ | _- No CMR evidence of myocardial damage was found in most (82%) of our MIS-C cohort, even though about 65% had depressed left ventricular ejection fraction at admission._  _- Acute myocarditis is a possible manifestation of MIS-C associated with COVID-19 with CMR evidence of myocardial necrosis in 18% of our cohort._  _- Independent of the presence of CMR signs of acute myocarditis, most children demonstrate rapid normalization of cardiac function._ |
| _Chakraborty A et al, 2022_  _Pediatric Cardiology (51)_ | _21_ | _Cross-sectional_ | _- 21 pediatric patients with MIS-C with a median age:of 11 years, who underwent CMR at median follow-up duration of 6 months._  _- At the peak of illness during admission, there were 95.2% patients with abnormal Troponin I and BNP._  _- Echocardiogram, 76.2% had LV systolic dysfunction and 9.5% had coronary ectasia, which all resolved by 6 months._  _- CMR: five patients (23.8%) with abnormal left atrial volume, one patient (4.8%) with an abnormal indexed LV end-diastolic volume, and three patients (15%) with abnormal LVEF. No evidence of myocardial edema in T2-weighted image sequence. Three patients with persistent late gadolinium enhancement (14.3%)._ | _- Limited studies have demonstrated that although systolic ventricular dysfunction function and coronary artery abnormalities almost always recover within 3–6 months, subtle abnormalities in diastolic function possibly reflective of myocardial injury persist in a subset of patients._  _-Follow-up CMR is a useful tool in diagnosing subtle myocardial abnormalities and guide necessity for future follow-up._ |
| _De Wolf RD et al, 2023_  _Frontiers in Pediatrics (52)_ | _36_ | _Prospective longitudinal (observational multicenter study)_ | _- 36 children with MIS-C and cardiac involvement between April 2020 and March 2022, with a median age of 10 years, and among them, 21 (58%) were girls._ | _- Late cardiac outcomes after MIS-C, if treated according to current guidelines, are excellent._  _- CMR does not show any myocardial scarring in children with a normal echocardiographic_ _left ventricular ejection fraction._  _- Subclinical myocardial damage can persist in the late term and further follow up seems appropriate in these patients._ |
| _Phirtskhalava S et al, 2023_  _Cureus (53)_ | _103_ | _Retrospective and prospective cohort_ | _- Georgian population diagnosed with MIS-C from Feb to March 2020, with a median age at admission: 7 years._  _- Most attention on children with cardiovascular complications (coronary artery abnormalities, arrhythmias, pericardial effusions, and valvular diseases)._  _-Exclusion criteria: preexisting comorbidities such as cardiac diseases._ | _- MIS-C commonly affects the cardiovascular system and leads to variable cardiac manifestations, most of which resolve with specific treatment courses and, fortunately, do not lead to major sequelae._  _-Systematic longer-term follow-up as well as standardized approaches to coronary artery imaging and interpretation are needed to provide clarity on the evolution of medium- and long-term cardiac outcomes in MIS-C._ |
| _Shah SS et al, 2023_  _Annals of Pediatric Cardiology (54)_ | _144_ | _Prospective, longitudinal,multi-center_ | _-Children < 18 years diagnosed with MIS-C (based on WHO criteria) referred for cardiac evaluation from June 1 to Nov 30, 2021._  _- Median age: 60 months._  _-All patients_ _have a positive test for SARS-CoV-2 by detection of serum antibodies or nucleic acid from a nasopharyngeal specimen._ | _- Cardiovascular manifestations are common in children with MIS-C._  _- Most children clinically recover from the acute illness and show significant early and intermediate recovery in terms of normalizing the myocardial and coronary abnormalities._  _-The long-term sequelae especially of coronary involvement need to be explored by follow-up studies._ |
| _Anagnostopoulou A et al, 2024_  _Pediatric Cardiology (55)_ | _25_ | _Prospective longitudinal, single-center_ | _-_ _Children aged 0–16 years old with a diagnosis of MIS-C (CDC and/or WHO criteria, who were admitted to the_  _Aghia Sophia” Children’s Hospital, Athens, Greece, hospital from January 01, 2021, to September 30, 2022_  _- Range age from 0 to 16 years with a mean age of 8.3 years and 64% were female._  _-_ _Exclusion criteria: pre-existing heart failure, CHD that required surgical intervention or any other form of severe cardiac disease._  _._ | _- The study highlighted that while most MIS-C-related cardiac abnormalities resolved over time, subclinical myocardial injuries persisted in a small subset of patients._  _-Left Ventricular Global Longitudinal Strain (LV-GLS) analysis was effective in identifying subtle myocardial dysfunction, even in cases with normal LVEF._ |
| _Leal GN et al, 2022_  _Microcirculation (56)_ | _6_ | _Cross- sectional_ | _-6 MIS-C patients admitted to a tertiary referral institution from July 2020 to July 2021-. 3 males, aged 9.3 (6.6– 15.7) years._  _-Time from admission to the follow- up visit: 6.05 (2– 10.3) months._  _- Although all patients were asymptomatic and LV EF was ≥55%, 43/102 (42.1%) LV segments showed_  _myocardial flow reserve <2.5_ | _- This is the first study to provide evidence that surviving MIS- C patients may present with subclinical impairment of myocardial microcirculation._  _- Dedicated pediatric follow- up programs must be set, to assess future cardiovascular outcomes in this population._  _-Segmental cardiac strain assessment by two- dimensional speckle-tracking echocardiography seems to be a useful technique for this purpose, given its large availability, cost effectiveness and good correlation with 13 N-ammonia PET-CT derived MFR._ |

CDC: Centers for Disease Control and Prevention; CHD_:_ Congenital Heart Disease; CRM: Cardiac Resonance Magnetic; EF: Ejection Fraction; ICU: Intensive Unite Care; KD: Kawasaki Disease; LV: left ventricle; MIS-C: Multisystem Inflammatory Syndrome in Children; WHO: World Health Organization; Ref.: Reference in the text
