# Supplementary material for: Cardiovascular abnormalities in multisystem inflammatory syndrome in children related to COVID-19
Source: Front Pediatr. 2026 Jan 5;13:1635723. doi: 10.3389/fped.2025.1635723 (PMC12812880; doi:10.3389/fped.2025.1635723)
Supplement: Supplementary Material Table S2 — Studies included in this review. [file Table2.docx]

| Section / Topic | Item | Checklist item | Status |
| --- | --- | --- | --- |
| TITLE | 1 | Identify the report as a systematic review. | ✔️ |
| ABSTRACT | 2 | See PRISMA 2020 for Abstracts. | ✔️ |
| INTRODUCTION |  |  |  |
| Rationale | 3 | Describe rationale for the review in the context of existing knowledge. | ✔️ |
| Objectives | 4 | Provide an explicit statement of the objective(s) or question(s) the review addresses. | ✔️ |
| METHODS |  |  |  |
| Eligibility criteria | 5 | Specify inclusion/exclusion criteria and how studies were grouped. | ✔️ |
| Information sources | 6 | Specify all databases/sources and date last searched. | ✔️ |
| Search strategy | 7 | Present full search strategies for all databases. | ✔️ |
| Selection process | 8 | Specify methods used to decide study eligibility. | ✔️ |
| Data collection process | 9 | Specify methods used to collect data from reports. | ✔️ |
| Data items | 10a | List and define all outcomes sought. | ✔️ |
|  | 10b | List and define other variables sought. | ✔️ |
| Study risk of bias assessment | 11 | Describe methods used to assess study quality or risk of bias. | ✔️ |
| Effect measures | 12 | Specify effect measures used. | N/A |
| Synthesis methods | 13a | Describe methods used to determine study eligibility. | ✔️ |
|  | 13b | Methods for data preparation. | ✔️ |
|  | 13c | Describe methods to synthesize results. | ✔️ |
|  | 13d | Describe methods to explore heterogeneity. | ✔️ |
|  | 13e | Describe methods to assess certainty. | ✔️ |
|  | 13f | Describe methods to assess reporting bias. | ✔️ |
| RESULTS |  |  |  |
| Study selection | 16a | Describe search and selection results; flow diagram. | ✔️ |
|  | 16b | Cite excluded studies with reasons. | ✔️ |
| Study characteristics | 17 | Cite characteristics of included studies. | ✔️ |
| Risk of bias – studies | 18 | Present risk of bias assessments. | ✔️ |
| Results of individual studies | 19 | Summarize study findings. | ✔️ |
| Synthesis results | 20a | Summarize synthesized findings. | ✔️ |
|  | 20b | Present statistical synthesis results. | N/A |
|  | 20c | Heterogeneity investigation. | ✔️ |
|  | 20d | Certainty of evidence. | ✔️ |
| DISCUSSION |  |  |  |
| Discussion | 23a | Interpret results in context. | ✔️ |
|  | 23b | Discuss limitations of evidence. | ✔️ |
|  | 23c | Discuss limitations of review process. | ✔️ |
|  | 23d | Discuss practice/research implications. | ✔️ |
| OTHER INFORMATION |  |  |  |
| Registration | 24 | Registration number (PROSPERO). | ✔️ |
| Protocol | 25 | Indicate whether a protocol exists. | ✔️ |
| Support | 26 | Describe financial support. | ✔️ – no funding |
| Competing interests | 27 | Declare conflicts of interest. | ✔️ |
| Availability of data & materials | 28 | State availability of data/materials. | ✔️ |
